# Nrf2/GPX4‐Dependent Ferroptosis Inhibition: The Central Mechanism Underpinning Germacrone‐Induced Cardioprotection in Myocardial Infarction

**DOI:** 10.1155/adpp/7893262

**Published:** 2026-05-26

**Authors:** Jiaxiang Liao, Zitian Wang, Zhou Huang, Jincheng Li, Jie Yang, Chunyun Fang, Dongling Huang, Fan Wang, Xueling Lu, Yiling Zhai, Wei Wang

**Affiliations:** ^1^ Department of Emergency Medicine, The First Affiliated Hospital of Guangxi Medical University, Nanning, 530021, Guangxi, China, gxmu.edu.cn; ^2^ Guangxi University Key Laboratory of Emergency Medicine, The First Affiliated Hospital of Guangxi Medical University, Nanning, 530021, Guangxi, China, gxmu.edu.cn; ^3^ Department of Emergency Medicine, Longgang Central Hospital of Shenzhen, Shenzhen, 518116, Guangdong, China; ^4^ Shenzhen Base of National Research Center for Emergency Medicine, Longgang Central Hospital of Shenzhen, Shenzhen, 518116, Guangdong, China

**Keywords:** ferroptosis, germacrone, GPX4, myocardial infarction, Nrf2

## Abstract

Myocardial infarction (MI) involves a pathological process in which its association with ferroptosis is notably pronounced, and targeted regulation of ferroptosis presents a promising approach for treating MI. This work delineates the molecular basis by which the natural sesquiterpene germacrone protects against cardiomyocyte ferroptosis following isoproterenol (ISO)‐induced MI. We established a murine model of ISO‐induced MI, paired with an injured H9c2 cardiomyocyte model in vitro. Mice in the experimental group received daily oral germacrone (25, 50, and 100 mg/kg) for 7 consecutive days. We profiled myocardial injury markers and key nuclear factor erythroid 2–related factor 2 (Nrf2)/glutathione peroxidase 4 (GPX4) axis proteins. The mechanism of action was confirmed by utilizing ML385, the Nrf2‐specific inhibitor. Germacrone significantly reduced ISO‐induced myocardial injury in mice, improving cardiac function and decreasing myocardial histopathological damage. Germacrone universally attenuated the elevation of myocardial injury and inflammatory markers in all experimental models. It also decreased the apoptosis rate of H9c2 cells. Germacrone inhibited myocardial ferroptosis and upregulated the abundance of pivotal regulatory proteins, including Nrf2, GPX4, and heme oxygenase‐1 (HO‐1), along with solute carrier family 7 member 11 (SLC7A11). ML385, an Nrf2 antagonist, can block germacrone’s protective effect against ISO‐induced myocardial ferroptosis and reverse its upregulation of these pathway proteins. These findings provide a new potential target and experimental foundation for MI treatment.

## 1. Introduction

Myocardial infarction (MI), the most prevalent form of ischemic heart disease in clinical practice, represents a formidable global public health challenge. Epidemiological data indicate that MI is responsible for approximately 8.9 million deaths annually worldwide, with its incidence continuing to exhibit a persistent upward trajectory [1]. The main pathophysiological process involves occlusion of the coronary artery, resulting in a sudden reduction in blood flow to the heart muscle and causing irreversible ischemic necrosis of cardiomyocytes [2]. Although percutaneous coronary intervention (PCI) is currently considered the gold standard for MI treatment, it can also cause additional damage to the heart during reperfusion, and timely access to primary PCI remains a major clinical challenge [3, 4]. Therefore, developing new agents to protect the myocardium has become a crucial focus in cardiovascular research.

Ferroptosis is a regulated type of cell death caused by excessive lipid peroxidation triggered by free iron and oxidative stress through the Fenton reaction following myocardial ischemia [5]. During MI, myocardial cell death occurs in various forms, including necrosis, apoptosis, autophagy, and ferroptosis [6]. Recent studies have revealed that ferroptosis may account for up to 30%–40% of cardiomyocyte loss during the acute phase of myocardial injury, with its relative contribution exceeding that of classical apoptosis at certain stages [7]. Collectively, these findings establish ferroptosis as a central pathogenic mechanism driving cardiomyocyte demise and subsequent myocardial tissue damage in the context of MI.

Nuclear factor erythroid 2–related factor 2 (Nrf2), a key regulator of the body’s antioxidant defense system, maintains cellular redox balance during oxidative stress and plays a vital role in inhibiting ferroptosis [8]. Accumulating studies have demonstrated that Nrf2 levels increase significantly after MI. Activation of the Nrf2/solute carrier family 7 member 11 (SLC7A11)/glutathione peroxidase 4 (GPX4) signaling pathway has been proven to effectively prevent ferroptosis [9]. Additionally, research indicates that muscone can reduce ferroptosis‐related damage following MI by affecting the Nrf2/GPX4 pathway [10]. Based on these findings, regulation of ferroptosis through the Nrf2/GPX4 pathway presents a promising target for MI treatment.

Germacrone is a naturally occurring sesquiterpene compound with the molecular formula C_15_H_22_O [11]. Sesquiterpenoids have demonstrated significant therapeutic potential across various fields, including antioxidation, anti‐inflammation, anticardiovascular disease, and anticancer activities [12]. Studies confirm that germacrone exhibits a wide range of biological activities, such as anti‐inflammatory, antioxidant, antithrombotic, antifibrotic, analgesic, lipid‐lowering, and platelet aggregation‐inhibiting properties [13]. It has been reported to improve neurological function after traumatic brain injury by suppressing neuroinflammation and oxidative stress [14]. In the present study, germacrone was selected for in‐depth investigation primarily due to its well‐documented potent anti‐inflammatory and antioxidant activities, which directly target the core pathological processes of MI. Furthermore, emerging evidence indicates that germacrone can modulate ferroptosis in a diabetic nephropathy model [15], providing a crucial clue for its potential analogous novel mechanism in cardiovascular diseases. Notably, although prior research has confirmed that germacrone can ameliorate cardiac remodeling via the PI3K/AKT pathway [16], whether it exerts its effects in MI through the regulation of ferroptosis, particularly via the Nrf2/GPX4 axis, remains entirely unexplored. Consequently, compared to other sesquiterpenoids or natural cardioprotective agents with relatively well‐elucidated mechanisms, research on germacrone’s role in ferroptosis‐targeted myocardial protection remains a gap in the field, rendering it an ideal candidate molecule for exploring this novel therapeutic avenue. Based on these findings, this study aims to explore whether germacrone inhibits myocardial ferroptosis via specific regulation of the Nrf2/GPX4 signaling cascade across in vitro H9c2 cells and in vivo isoproterenol (ISO)‐induced MI models.

## 2. Materials and Methods

### 2.1. Reagents

The commercial source for ISO (CAS 51‐30‐9) and the Nrf2 inhibitor ML385 (CAS 846557‐71‐9) was Aladdin Biotechnology Co., Ltd. (Shanghai, China). Germacrone (purity > 99%, CAS 6902‐91‐6) was sourced from Chengdu Gelipu Biological Technology Co., Ltd. (Chengdu, China). Primary antibodies targeting Nrf2 (cat. no. 16396‐1‐AP), heme oxygenase‐1 (HO‐1) (cat. no. 10701‐1‐AP), SLC7A11 (cat. no. 26864‐1‐AP), and GPX4 (cat. no. 67763‐1‐Ig) were obtained from Wuhan Sanying Biotechnology Co., Ltd. (Wuhan, China). Superoxide dismutase (SOD, cat. no. A001‐3), reduced glutathione (GSH, cat. no. A006‐2‐1), Cell Counting Kit‐8 (CCK‐8, cat. no. G021‐1), and malondialdehyde (MDA, cat. no. A003‐1) were obtained from Nanjing Jiancheng Bioengineering Institute (Nanjing, China). Assays for intracellular Fe^2+^ and reactive oxygen species (ROS) were conducted using commercial kits from distinct suppliers: Fe^2+^ levels were quantified with BC5415 kits (Beijing Solarbio Technology), while ROS detection employed BL714A kits (Beijing Lanjieke Technology). Quantification of circulating inflammatory mediators and myocardial injury biomarkers was performed using vendor‐supplied ELISA kits (Shanghai Fankewei Biological Technology Co., Ltd., Shanghai, China). The analyzed targets included interleukin‐6 (IL‐6; cat. no. F30717‐A), tumor necrosis factor‐α (TNF‐α; F2132‐A), interleukin‐1β (IL‐1β; F30626‐A), creatine kinase isoenzyme (CK‐MB; F2801‐A), lactate dehydrogenase (LDH; F9207‐A), and cardiac troponin I (cTnI; F2379‐A).

#### 2.1.1. Compound Source and Quality Control

Germacrone (CAS 6902‐91‐6) was sourced from Chengdu Gelipu Biological Technology Co., Ltd. (Chengdu, China). Its chemical purity was verified by the supplier using high‐performance liquid chromatography (HPLC) under the following conditions: Kromasil 100‐5‐C18 column (4.6 × 250 mm), a mobile phase consisting of acetonitrile and 0.1% phosphoric acid solution with gradient elution, a detection wavelength set at 216 nm, and a column temperature maintained at 30°C. Analysis indicated that the main germacrone peak area accounted for > 99.91% (Supporting Figure S1), confirming a purity suitable for the experimental requirements. All germacrone used in the experiments was aliquoted, stored in light‐protected, sealed containers at 2°C–8°C, and showed no visible signs of degradation or changes in physical properties throughout the study period, indicating good stability under these storage conditions.

### 2.2. H9c2 Cell Culture and Intervention Protocol

H9c2 rat cardiac myoblasts were purchased from ATCC (USA) and authenticated before use. For experimental procedures, P8 cells were seeded into six‐well plates at 5 × 10^5^ cells/well to achieve approximately 70% confluency at treatment onset. Cells were maintained in high‐glucose DMEM supplemented with 10% fetal bovine serum, 100 U/mL penicillin, and 100 μg/mL streptomycin and were incubated at 37°C under 5% CO_2_ with humidification. For drug intervention, cells were preincubated with gradient germacrone (25, 50, and 100 μM) or 5 μM ML385 for 24 h. After medium aspiration, 100 μM ISO was added for another 24 h to induce injury; cells treated with an equal volume of dimethyl sulfoxide (DMSO) served as a negative control.

### 2.3. Animal Experiment

All experimental protocols received official ethical approval from the Institutional Review Board of the First Affiliated Hospital of Guangxi Medical University (Authorization Code: 2025‐D0457) before commencement of the study. The experimental cohort consisted of 60 male C57BL/6 mice (aged 6–8 weeks; initial body weight, 22–26 g) obtained from the Laboratory Animal Center of Guangxi Medical University. Animals were randomly allocated into six treatment groups (*n* = 10 per group): a control group, an ISO‐induced MI group (100 mg/kg/day), three MI groups receiving graded doses of germacrone (25, 50, or 100 mg/kg/day), and a final group receiving high‐dose germacrone combined with the Nrf2 inhibitor ML385. Corn oil containing 0.4% DMSO was utilized as the vehicle. Germacrone was dissolved in this vehicle for oral administration, a formulation strategy necessitated by its high lipophilicity and extremely low aqueous solubility, which precludes the preparation of a high‐dose aqueous solution. This method is commonly employed in pharmacological studies of sesquiterpenoids. The dose of ISO was determined based on a previous study [17]. For the control group, each mouse received 0.5 mL/20 g of the vehicle via oral gavage each morning from Days 1 to 7 and 0.1 mL/20 g of normal saline via subcutaneous injection each afternoon from Days 6 to 7. Mice in the ISO‐induced MI group received the same vehicle volume (0.5 mL/20 g) via gavage each morning from Days 1 to 7. From Days 6 to 7, mice in this group received daily afternoon subcutaneous injections of ISO at 100 mg/kg/day (injection volume: 0.1 mL per 20 g of body mass). In each of the three MI + germacrone dose groups, mice received germacrone (at the respective dose in 0.5 mL/20 g vehicle) via gavage each morning from Days 1 to 7, alongside the same ISO injection regimen (100 mg/kg/day, afternoons, Days 6–7) as the MI group. In the MI + high‐dose germacrone + ML385 group, mice received germacrone (100 mg/kg/day in 0.5 mL/20 g vehicle) via gavage each morning from Days 1 to 7, followed by an intraperitoneal injection of ML385 at 30 mg/kg. On the afternoons of Days 6 and 7, these mice also received the same ISO injections as described for the ISO‐induced MI group. All mice underwent echocardiography 24 h after the final ISO injection, followed immediately by euthanasia and collection of cardiac tissues.

### 2.4. Echocardiography Evaluation

Prior to echocardiographic examination, mice were anesthetized via inhalation of 3%–4% isoflurane for induction, followed by maintenance at 1%–2%. Noninvasive echocardiography was performed on anesthetized mice using the D860LAB system (Feinno, China) to obtain standard cardiac parameters: left ventricular end‐diastolic volume (LVEDV), ejection fraction (EF), left ventricular end‐systolic volume (LVESV), and fractional shortening (FS).

### 2.5. HE Staining

Euthanasia was performed by increasing the isoflurane concentration to over 5% and maintaining exposure for at least 2 min after the examination. Cardiac tissues were subsequently collected for histopathological evaluation. The processing protocol included fixation in 4% paraformaldehyde, paraffin embedding, and microtome sectioning at 4 μm. Following dewaxing with xylene, the sections were stained with hematoxylin and eosin, and myocardial architecture was examined under a light microscope.

### 2.6. CCK‐8 Assay

H9c2 cardiomyocytes were seeded into 96‐well culture plates at a density of 1 × 10^4^ cells per well and maintained for 24 h to ensure complete adhesion prior to experimental manipulations. Subsequently, cells were exposed to concentration gradients of ISO (1.56–200 μM) and germacrone (1.56–200 μM), with 20 μM valsartan serving as a positive control, for an additional 24 h. The concentration range of germacrone (1.56–200 μM) was determined based on preliminary pilot experiments and existing literature reporting its cellular effects [18]. Cellular viability was quantified with the Cell Counting Kit‐8 according to the supplier’s recommended procedures. Specifically, 10 μL of CCK‐8 solution was introduced into each culture well, with subsequent incubation for 2 h at 37°C. Following 10 min of gentle agitation at ambient temperature, optical density readings were obtained at 450 nm employing a microplate reader. All standards and samples were analyzed in duplicate (or triplicate), and the mean value was used for subsequent analysis.

### 2.7. ELISA Detection

Precoated microwells containing specific antibodies against CK‐MB, LDH, cTnI, IL‐1β, IL‐6, and TNF‐α received sequential additions of processed samples (cell cultures and myocardial tissues), reference standards, and horseradish peroxidase (HRP)–conjugated detection antibodies according to the established protocol. After incubation, the wells were thoroughly washed. The chromogenic substrate TMB was then added to develop color. Under the catalytic action of peroxidase, TMB initially turned blue and was later converted to yellow after acid treatment. Absorbance measurements at 450 nm were acquired using a full‐spectrum microplate detection system.

### 2.8. TUNEL Assay

Following a 30‐min fixation with 4% paraformaldehyde, TUNEL staining was conducted on treated H9c2 cardiomyocytes using standardized commercial protocols. The enzymatic reaction, utilizing a mixture of TdT and fluorescent labeling solution, was carried out at 37°C for 60 min under dark conditions.

### 2.9. Determination of Fe^2+^, MDA, GSH, and SOD Levels

The detection of target indicators (Fe^2+^, MDA, GSH, and SOD) in cells and cardiac tissues was performed strictly in accordance with the operational guidelines of the corresponding assay kits, and their respective levels were measured accordingly.

### 2.10. ROS Staining

H9c2 cells were gently shaken and incubated with dichlorodihydrofluorescein diacetate (DCFH‐DA) working solution for 20 min at ambient temperature in the dark, and subsequent nuclear counterstaining was performed using Hoechst 33,342 (2′‐(4‐ethoxyphenyl)‐5‐(4‐methyl‐1‐piperazinyl)‐2,5′‐bi‐1H‐benzimidazole trihydrochloride), applying a 20‐min incubation under light‐protected conditions at ambient temperature. Following this step, cellular specimens underwent three sequential washing cycles, and ROS production was detected under a fluorescence microscope using excitation/emission wavelengths of 545/590 nm. The relative ROS production was calculated as the ratio of red to blue fluorescence intensity.

### 2.11. Mitochondrial Superoxide ROS (mtROS) Detection

H9c2 cells were gently shaken and incubated with 1 μM MitoSOX Red probe working solution for an incubation duration of 20 min at room temperature in the dark, after which nuclei were stained using Hoechst 33,342 (a specific nuclear fluorescent dye) at room temperature in the dark for 20 min. Following this step, cellular specimens underwent three sequential washing cycles, and the production of mtROS was detected under a fluorescence microscope with excitation/emission wavelengths of 510/580 nm. The relative mtROS production was calculated as the ratio of red to blue fluorescence intensity.

### 2.12. Protein Isolation From Biological Specimens and Immunoblot Analysis

Protein lysates were prepared from cardiac tissues and H9c2 cardiomyocytes using RIPA buffer containing a protease inhibitor cocktail. After determining protein concentrations using the Bradford method, equivalent protein quantities were separated by SDS–PAGE and electrotransferred onto PVDF membranes. For immunoblot analysis, membranes were probed with specific primary antibodies against Nrf2, HO‐1, GPX4, SLC7A11, and β‐actin (4°C, overnight) and then exposed to corresponding HRP‐linked secondary antibodies (room temperature, 1 h). Signal visualization was achieved through enhanced chemiluminescence detection.

### 2.13. Statistical Analysis

Results from at least three biologically independent replicates are expressed as mean ± standard deviation. Data visualization and graphical representation were generated using GraphPad Prism 9.0 (La Jolla, CA). Statistical comparisons among experimental groups were conducted by one‐way analysis of variance in SPSS 23.0 (Chicago, IL), establishing statistical significance at *p* < 0.05.

## 3. Results

### 3.1. Germacrone Inhibits MI Caused by ISO

ISO is widely acknowledged as a standard agent for establishing MI models in animal experiments. This investigation established a murine MI model through 2 consecutive days of subcutaneous injections of ISO (100 mg/kg/day), subsequently evaluating the cardioprotective properties of germacrone within this experimental system. Echocardiography showed a decrease in EF and FS in the MI group, while LVEDV and LVESV increased. Germacrone administration significantly improved EF and FS and decreased LVEDV and LVESV in a concentration‐dependent fashion (Figures 1(a), 1(b), 1(c), 1(d), and 1(e)). The cardioprotective pharmacological activity of germacrone was reversed by the Nrf2 inhibitor ML385. HE staining revealed that, compared with the control specimens, the ISO‐administered animals had disorganized myocardial architecture, extensive myofibril disruption, and infiltrative accumulation of neutrophils and macrophages in the myocardium, along with interstitial edema and cell necrosis (Figure 1(f)). In the germacrone pretreatment group, the myofibrillary structure gradually recovered; inflammation was reduced, and interstitial swelling and cell necrosis were notably decreased. These findings demonstrate that ML385 exacerbates myocardial damage.

FIGURE 1Germacrone reduces myocardial injury caused by ISO. (a–e) Echocardiographic assessment of murine cardiac function evaluated germacrone’s effects on key hemodynamic parameters: left ventricular end‐diastolic volume (LVEDV), end‐systolic volume (LVESV), ejection fraction (EF), and fractional shortening (FS). Values represent mean ± SD (*n* = 8). ^##^ *p* < 0.001 vs. control; ^∗∗∗^ *p* < 0.001 vs. ISO group; ^&&&^ *p* < 0.001 vs. high‐dose germacrone group. (f) Representative H&E‐stained myocardial sections (*n* = 5). Scale bar: 50 μm.

**Figure:**
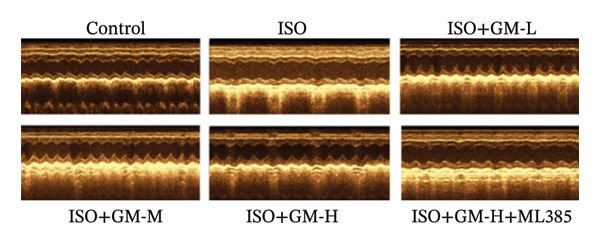
(a)

**Figure:**
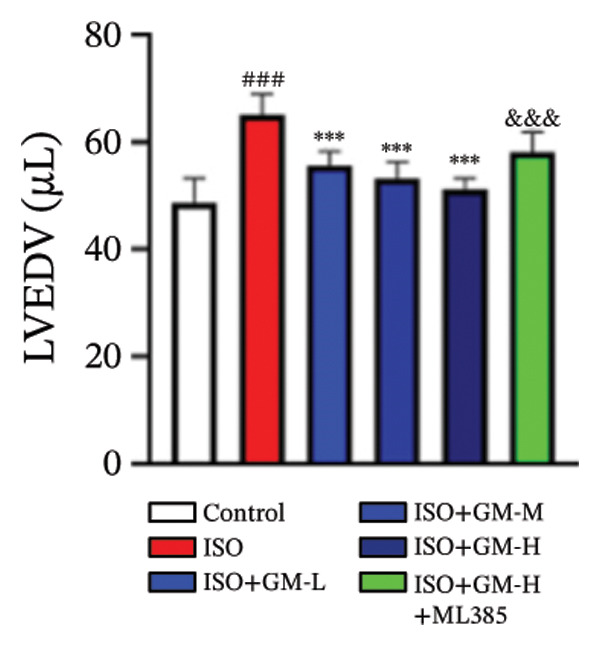
(b)

**Figure:**
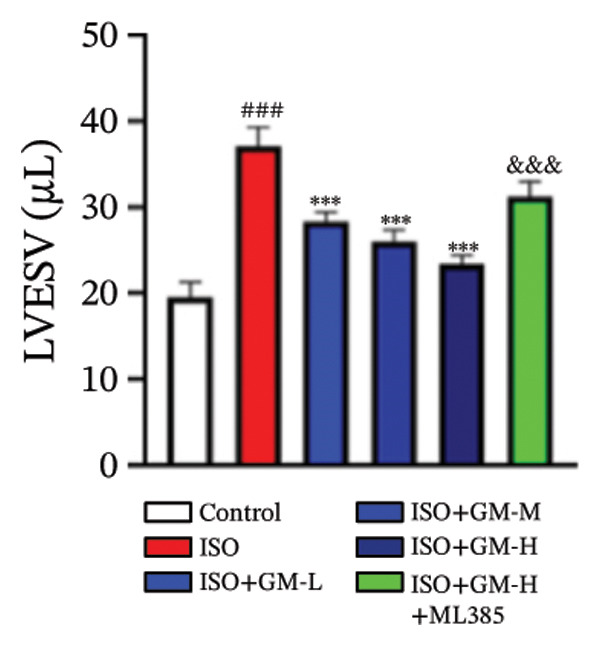
(c)

**Figure:**
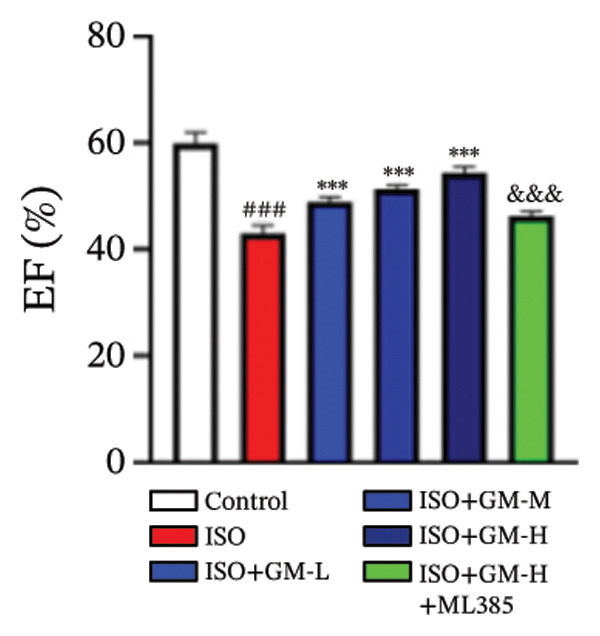
(d)

**Figure:**
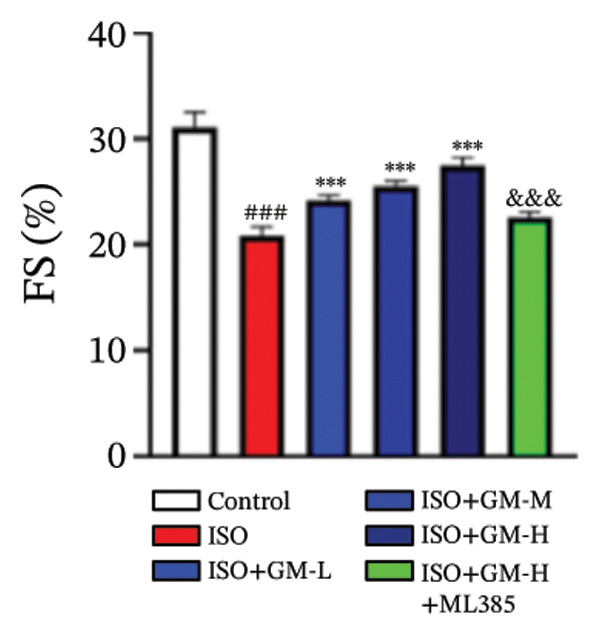
(e)

**Figure:**
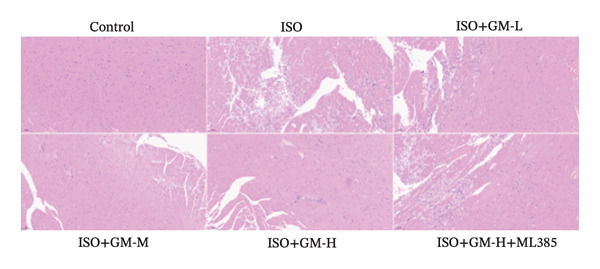
(f)

At the cellular level, after treating H9c2 cells with ISO at various concentrations, a 100 μM ISO concentration was found to reduce cell survival to about 50% of the normal level. As a result, an in vitro myocardial injury model was established at this concentration (Figure 2(a)). Next, the toxicity of germacrone was assessed using the CCK‐8 method: It was nontoxic below 100 μM, but cell viability decreased and toxicity appeared at 200 μM (Figure 2(b)). The following experiments used doses of 25, 50, and 100 μM. Further studies confirmed that germacrone exerted a marked capacity to elevate the survival rate of H9c2 cells injured by ISO, and this protective effect exhibited a clear dose‐dependent pattern, with 100 μM showing an effect comparable to valsartan (Figure 2(c)). These findings demonstrate that germacrone confers a protective role against myocardial cell injury induced by ISO. CK‐MB, LDH, and cTnI are widely recognized as classical markers for assessing cardiac injury. Quantitative analysis of murine cardiac tissue and H9c2 cardiomyocytes using standardized commercial assays revealed that ISO challenge markedly elevated concentrations of myocardial damage biomarkers (CK‐MB, LDH, and cTnI) relative to control measurements. Germacrone was found to decrease these injury markers, including a dose‐dependent reduction of ISO‐induced elevated LDH in mouse myocardial tissue, as well as cTnI and LDH in H9c2 cells. It has been shown that ML385 can reverse the reduction caused by germacrone on these myocardial enzyme indicators (Figures 2(d), 2(e), 2(f), 2(g), 2(h), and 2(i)).

FIGURE 2Germacrone inhibits ISO‐induced myocardial injury. (a) ISO‐mediated cytotoxicity in H9c2 cardiomyoblasts (*n* = 4). ^∗∗∗^ *p* < 0.001 vs. control. (b) Germacrone cytotoxicity profile in H9c2 cells (*n* = 4). ^∗∗∗^ *p* < 0.001 vs. control. (c) Germacrone cytoprotection in ISO‐injured H9c2 cells (*n* = 4). ^###^ *p* < 0.001 vs. control; ^∗∗^ *p* < 0.01, ^∗∗∗^ *p* < 0.001 vs. ISO group. (d–f) Myocardial enzyme profiles (CK‐MB, LDH, and cTnI) in murine cardiac tissue (mean ± SD, *n* = 6). ^###^ *p* < 0.001 vs. control; ^∗∗^ *p* < 0.01, ^∗∗∗^ *p* < 0.001 vs. ISO group; ^&&&^ *p* < 0.001 vs. high‐dose germacrone. (g–i) Cardiomyocyte enzyme release (CK‐MB, LDH, and cTnI) in H9c2 cells (mean ± SD, *n* = 6). ^###^ *p* < 0.001 vs. control; ^∗∗∗^ *p* < 0.001 vs. ISO group; ^&&&^ *p* < 0.001 vs. high‐dose germacrone.

**Figure:**
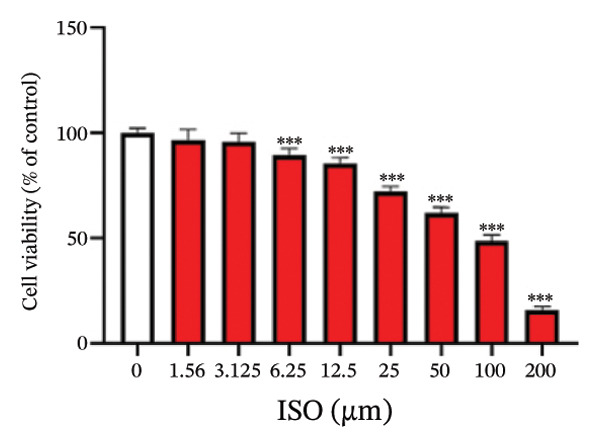
(a)

**Figure:**
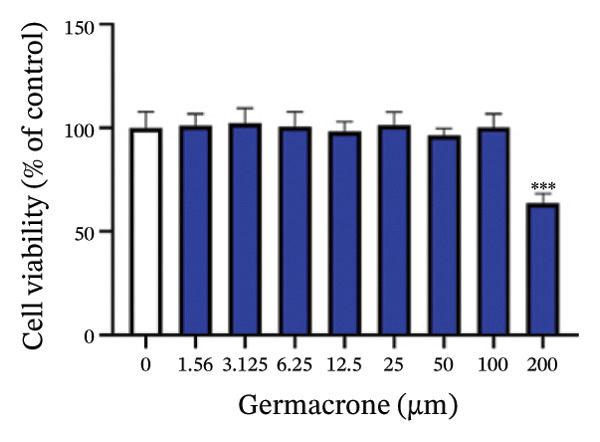
(b)

**Figure:**
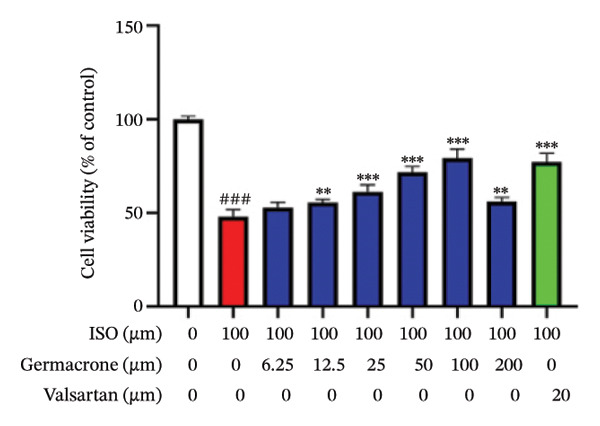
(c)

**Figure:**
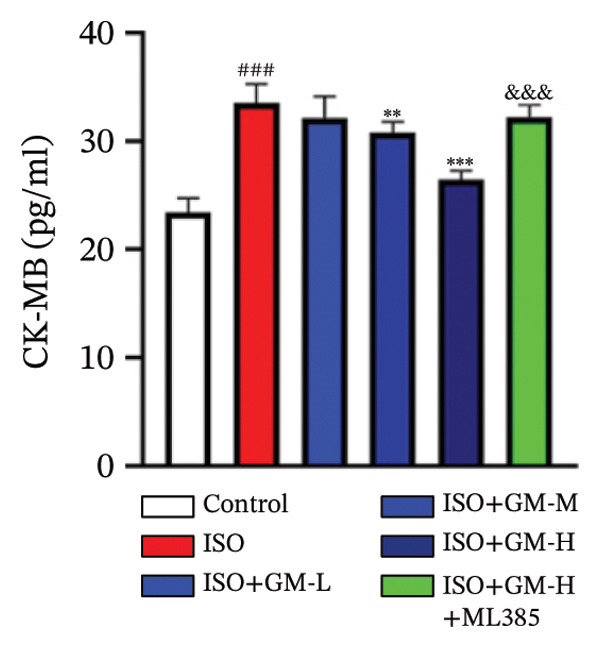
(d)

**Figure:**
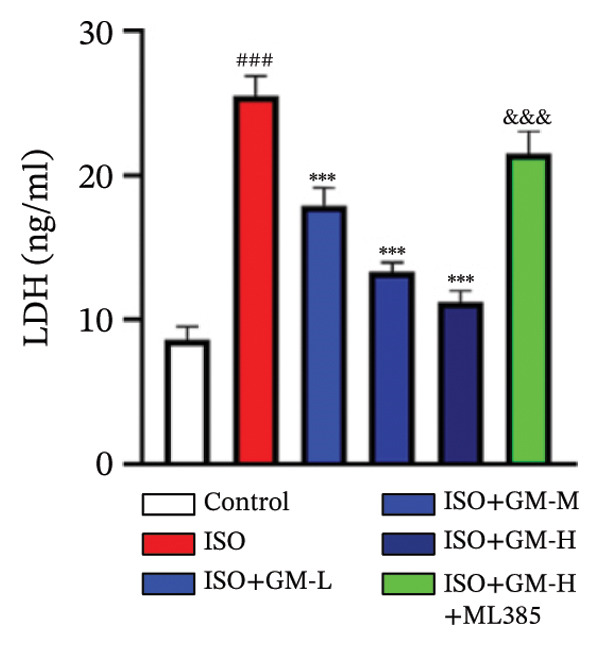
(e)

**Figure:**
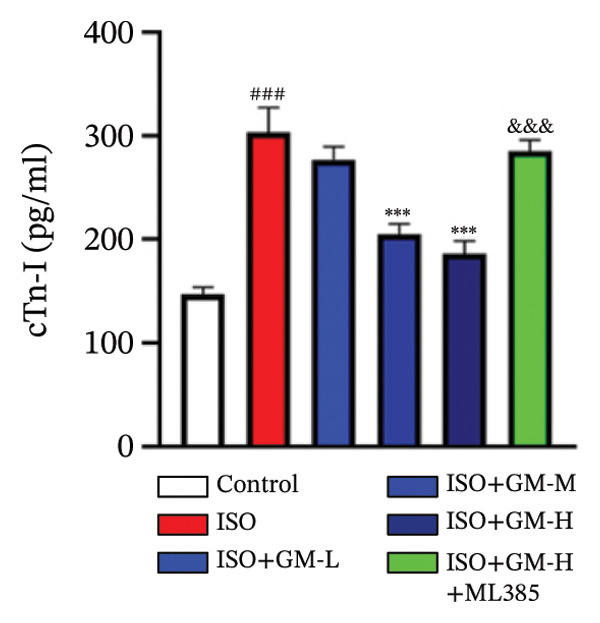
(f)

**Figure:**
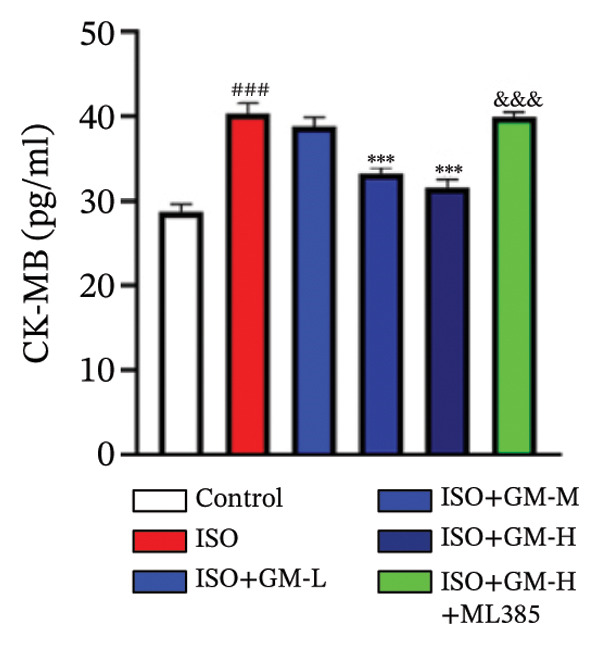
(g)

**Figure:**
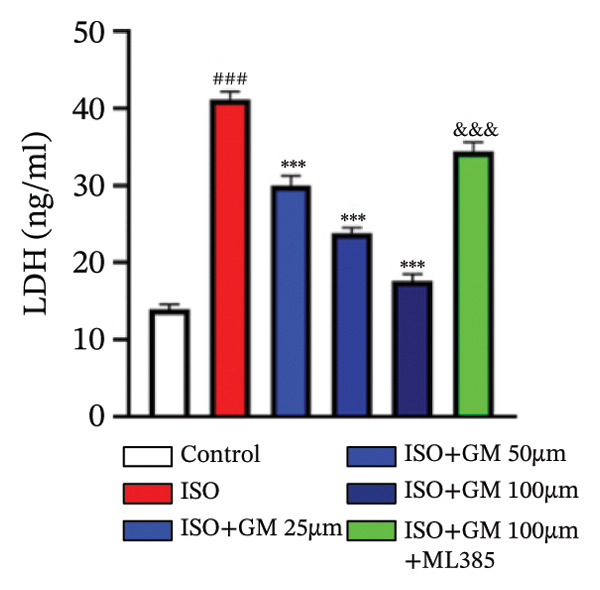
(h)

**Figure:**
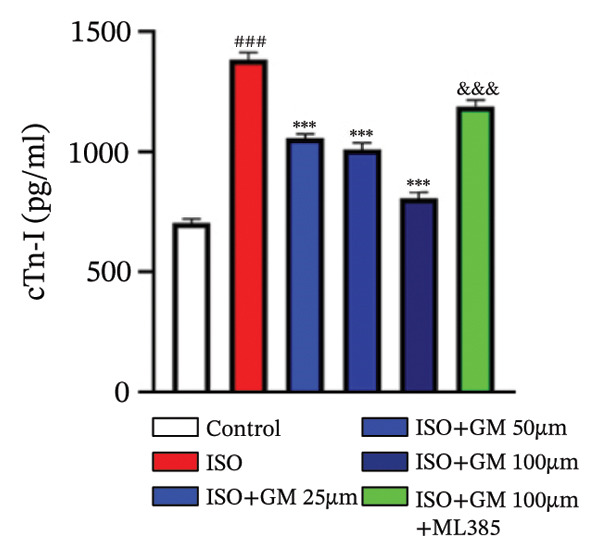
(i)

### 3.2. Germacrone Inhibits Inflammation and Apoptosis Caused by ISO

IL‐6, TNF‐α, and IL‐1β act as key drivers of inflammation in the context of cardiac injury. Systematic quantification with standardized assay kits confirmed that ISO challenge markedly increased concentrations of proinflammatory mediators (IL‐6, TNF‐α, and IL‐1β) in both murine myocardial tissue and H9c2 cardiomyocytes compared to control conditions. Germacrone potently suppressed IL‐1β in ISO‐injured myocardium and reduced both IL‐6 and IL‐1β in cardiomyocytes, with a clear dose–response relationship. This anti‐inflammatory effect of germacrone was reduced by ML385, which reversed the downregulation of these cytokines (Figures 3(a), 3(b), 3(c), 3(d), 3(e), and 3(f)). Furthermore, TUNEL staining data demonstrated that germacrone markedly suppressed ISO‐induced H9c2 cell apoptosis in a dose‐dependent fashion, thus lowering the apoptosis rate. However, ML385 partially offset this protective effect, leading to an increased rate of apoptosis (Figures 3(g), 3(h)).

FIGURE 3Germacrone inhibits inflammation and apoptosis caused by ISO. (a–c) Murine myocardial tissues were subjected to quantitative analysis of proinflammatory cytokine concentrations (IL‐6, TNF‐α, and IL‐1β). Data represent mean ± SD (*n* = 6). ^###^ *p* < 0.001 vs. control; ^∗^ *p* < 0.05, ^∗∗^ *p* < 0.01, ^∗∗∗^ *p* < 0.001 vs. ISO; ^&&&^ *p* < 0.001 vs. high‐dose germacrone. (d–f) Cytokine secretion profiles (IL‐6, TNF‐α, and IL‐1β) in H9c2 cardiomyocytes. Values are shown as mean ± SD (*n* = 6). ^###^ *p* < 0.001 vs. control; ^∗∗∗^ *p* < 0.001 vs. ISO; ^&&&^ *p* < 0.001 vs. high‐dose germacrone. (g–h) Apoptotic cell death was assessed by TUNEL staining in H9c2 cells, with quantitative analysis of apoptosis rates (*n* = 5). ^###^ *p* < 0.001 vs. control; ^∗∗∗^ *p* < 0.001 vs. ISO; ^&&&^ *p* < 0.001 vs. high‐dose germacrone. Scale bar: 100 μm.

**Figure:**
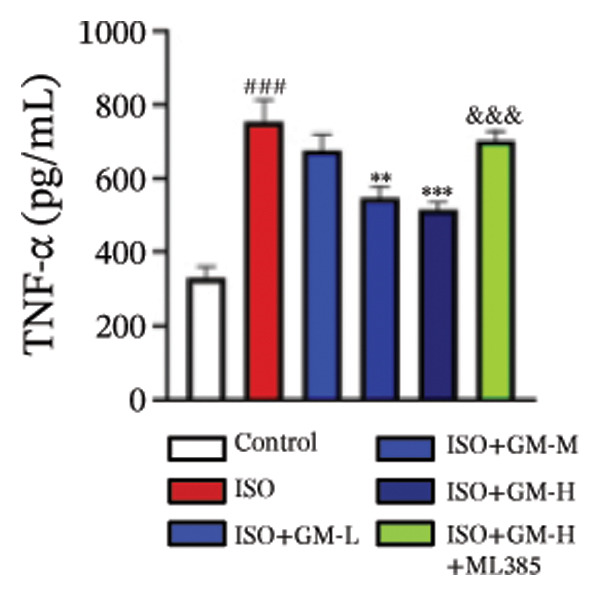
(a)

**Figure:**
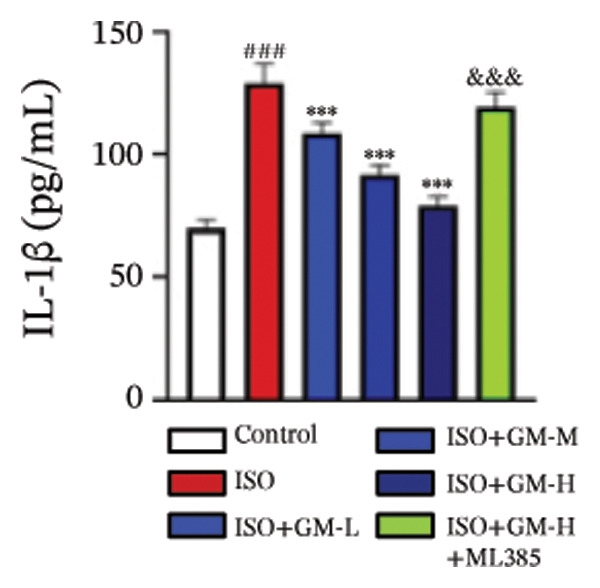
(b)

**Figure:**
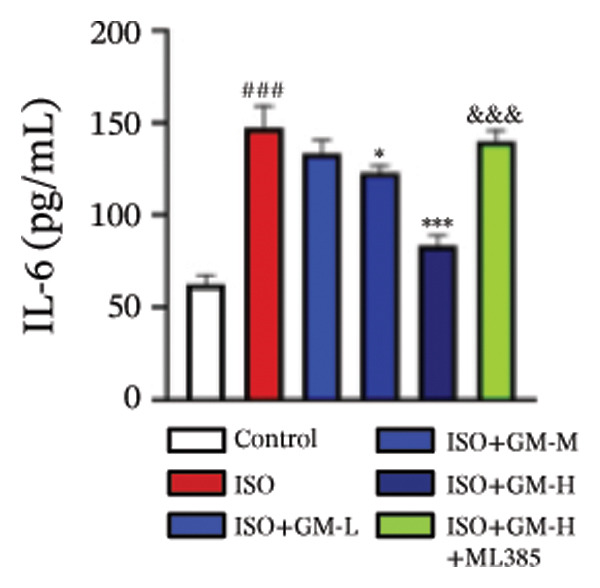
(c)

**Figure:**
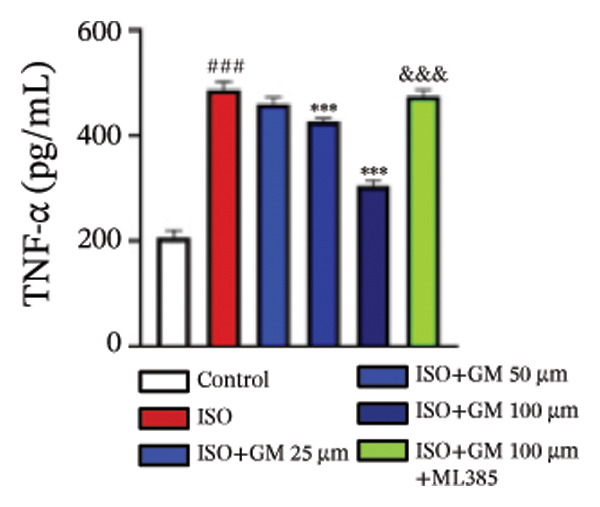
(d)

**Figure:**
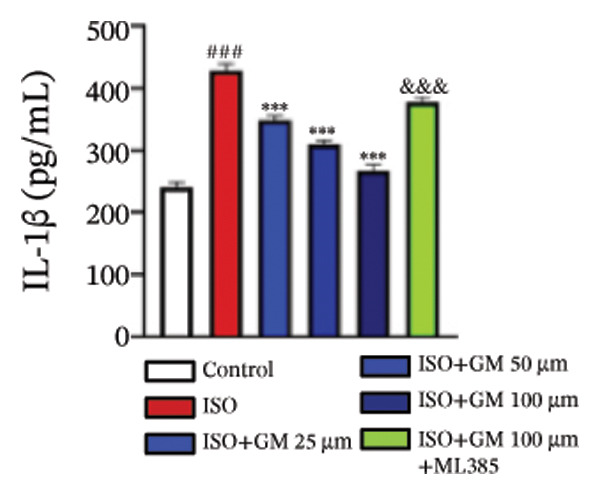
(e)

**Figure:**
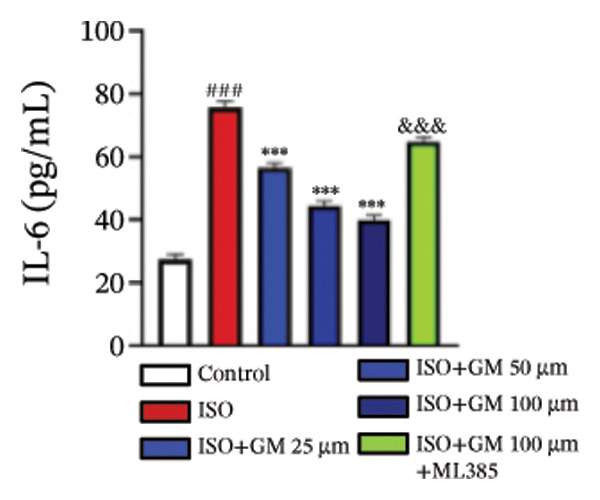
(f)

**Figure:**
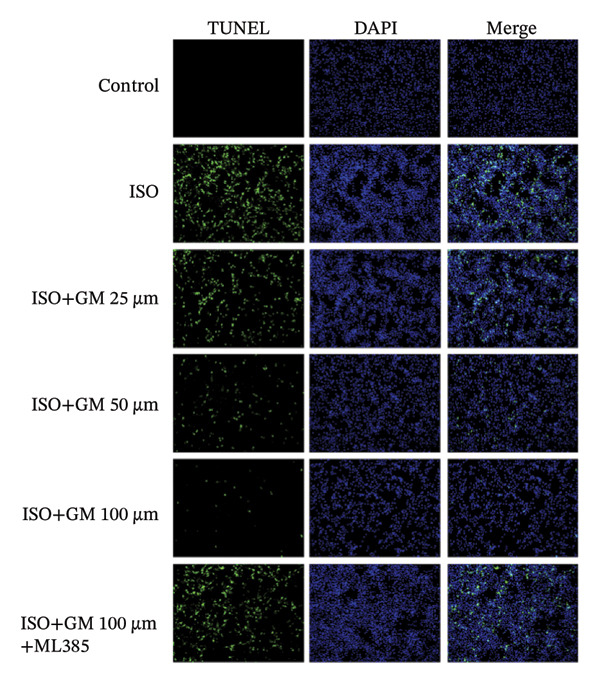
(g)

**Figure:**
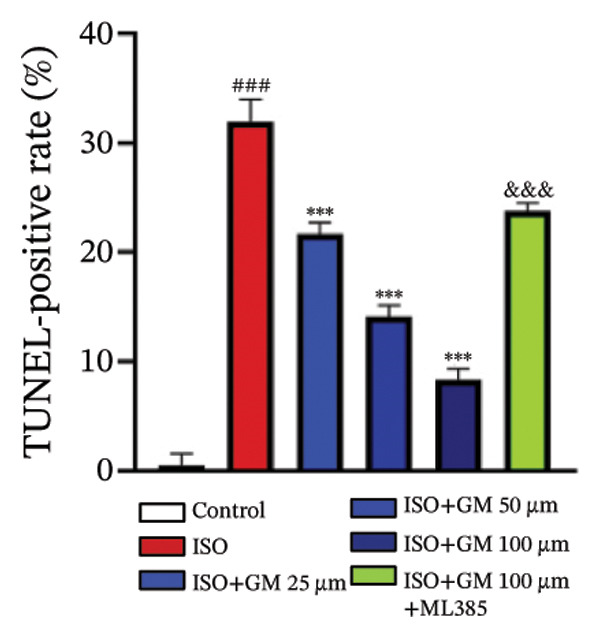
(h)

### 3.3. Germacrone Inhibits ISO‐Induced Myocardial Ferroptosis

To investigate the antiferroptotic potential of germacrone in alleviating ISO‐induced myocardial injury, we quantified key biomarkers of ferroptosis (Fe^2+^, MDA, GSH, and SOD) in both murine cardiac tissue and H9c2 cells using standardized commercial kits. Relative to the control group, our findings demonstrated that Fe^2+^ and MDA levels increased after ISO induction, while GSH and SOD levels decreased. Germacrone was able to inhibit the rise in MDA and Fe^2+^ and reverse the decrease in GSH and SOD caused by ISO. However, ML385 caused these marker levels to approach those of the ISO‐induced group (Figures 4(a), 4(b), 4(c), 4(d), 4(e), 4(f), 4(g), and 4(h)). ROS and mtROS are also indicators of ferroptosis. Germacrone significantly reduced the production of both ROS and mtROS after ISO stimulation in a dose‐dependent manner, while ML385 reversed this effect by increasing their production (Figures 5(a), 5(b), 5(c), and 5(d)).

FIGURE 4Germacrone inhibits ISO‐induced myocardial ferroptosis. (a–d) Murine cardiac tissue analysis of ferroptosis‐related biomarkers (Fe^2+^, MDA, GSH, and SOD). Data are expressed as mean ± SD (*n* = 6). ^###^ *p* < 0.001 vs. control; ^∗^ *p* < 0.05, ^∗∗^ *p* < 0.01, ^∗∗∗^ *p* < 0.001 vs. ISO; ^&&&^ *p* < 0.001 vs. high‐dose germacrone. (e–h) Assessment of ferroptosis parameters (Fe^2+^, MDA, GSH, and SOD) in H9c2 cardiomyocytes. Values are shown as mean ± SD (*n* = 6). ^###^ *p* < 0.001 vs. control; ^∗∗∗^ *p* < 0.001 vs. ISO; ^&&&^ *p* < 0.001 vs. high‐dose germacrone.

**Figure:**
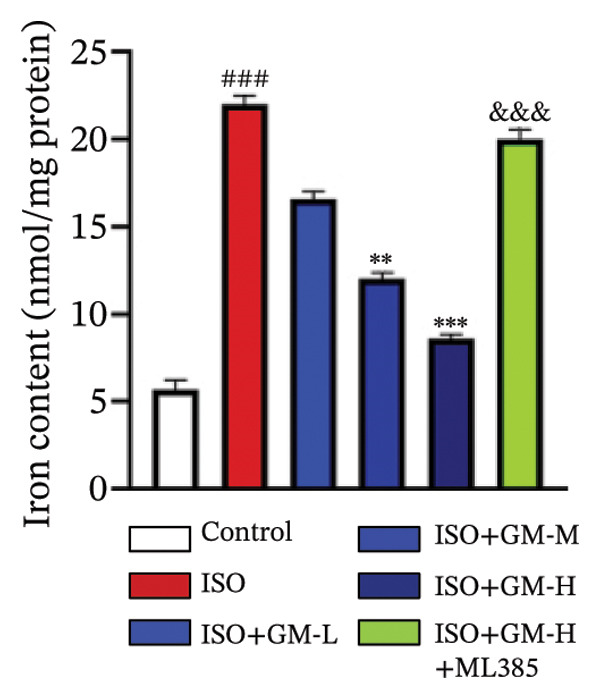
(a)

**Figure:**
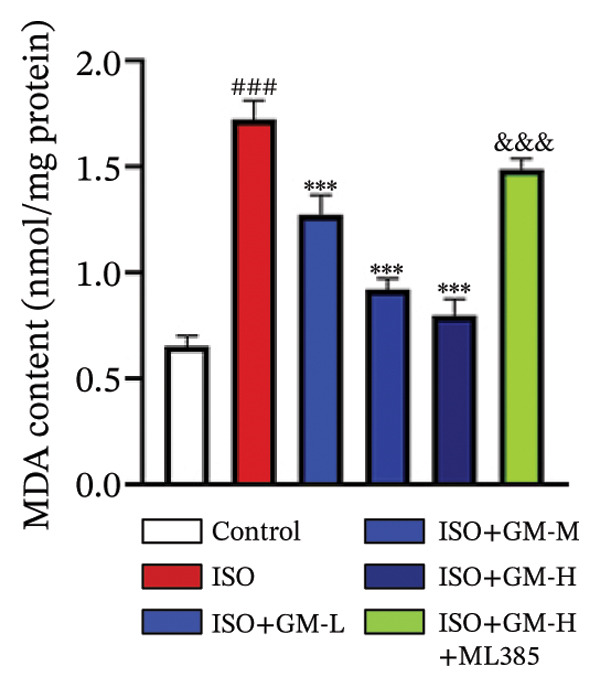
(b)

**Figure:**
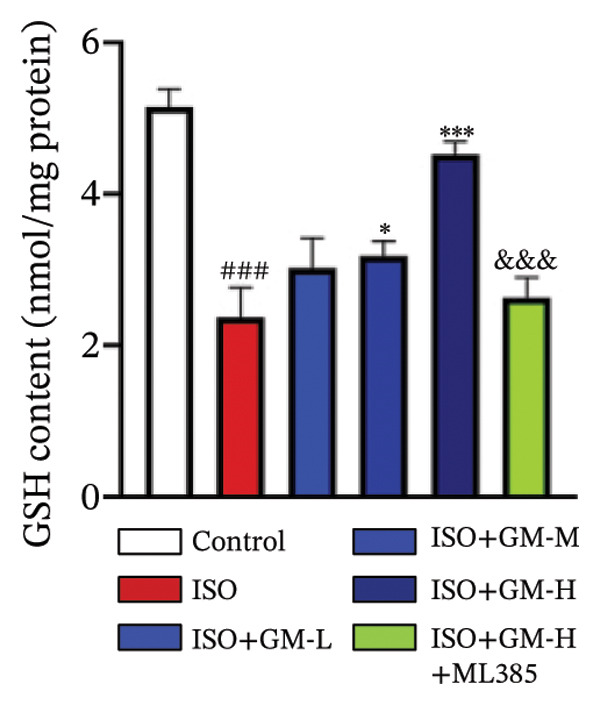
(c)

**Figure:**
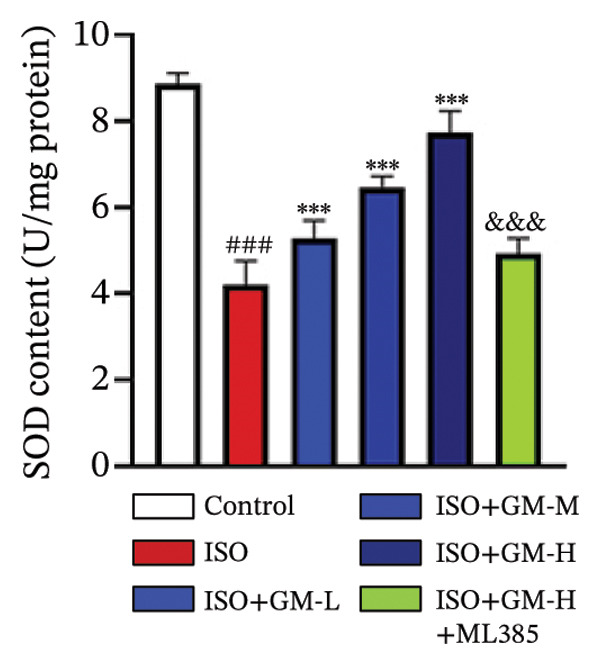
(d)

**Figure:**
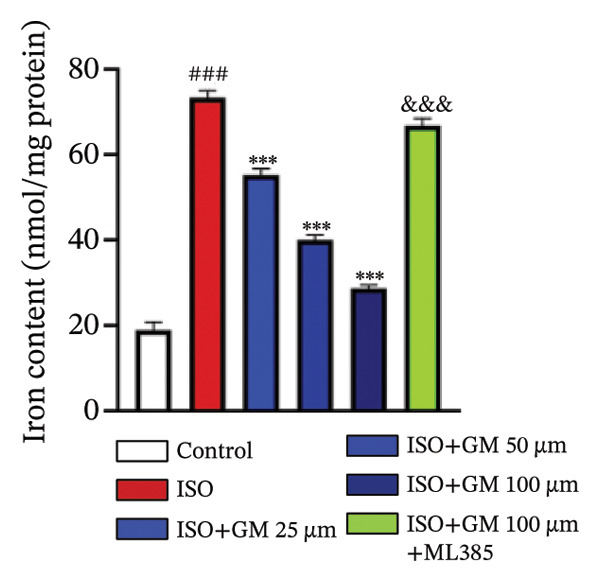
(e)

**Figure:**
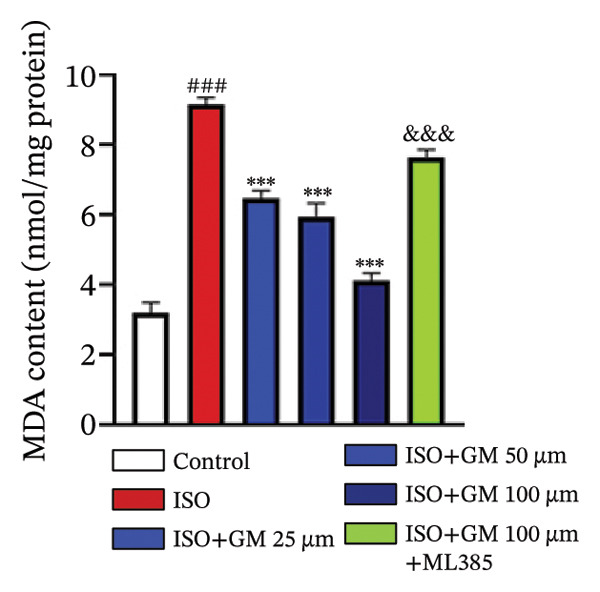
(f)

**Figure:**
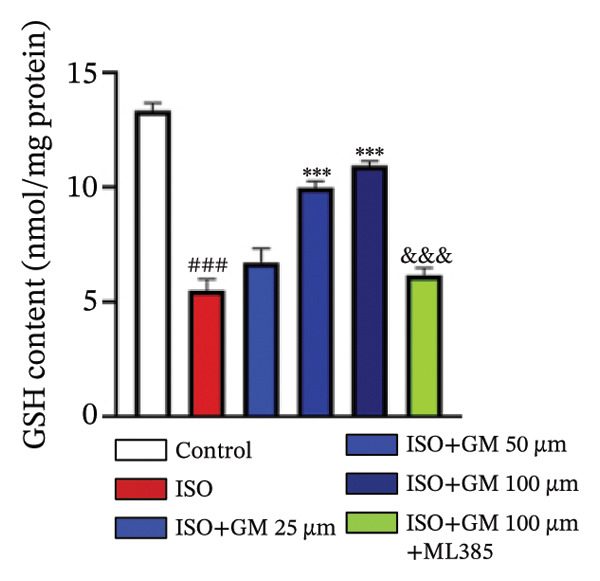
(g)

**Figure:**
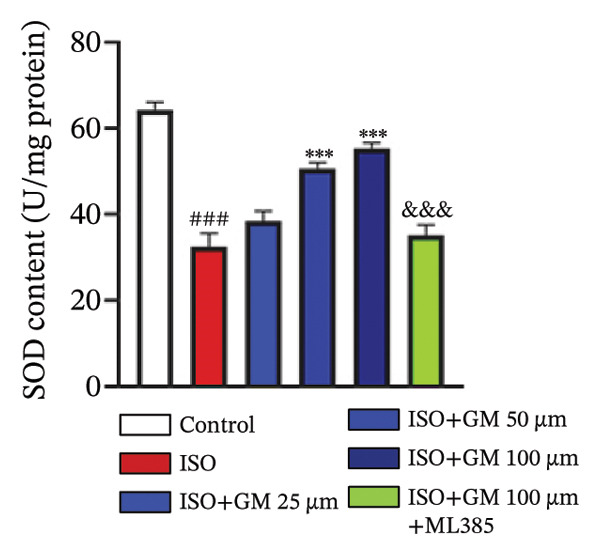
(h)

FIGURE 5Germacrone inhibits ISO‐induced myocardial ferroptosis. (a and c) Quantitative analysis of germacrone’s effects on intracellular ROS in ISO‐stimulated H9c2 cardiomyocytes (*n* = 5). ^###^ *p* < 0.001 vs. control; ^∗∗∗^ *p* < 0.001 vs. ISO; ^&&&^ *p* < 0.001 vs. high‐dose germacrone. Scale bar = 100 μm. (b and d) Mitochondrial ROS modulation by germacrone in ISO‐injured H9c2 cells (*n* = 5). ^###^ *p* < 0.001 vs. control; ^∗∗∗^ *p* < 0.001 vs. ISO; ^&&&^ *p* < 0.001 vs. high‐dose germacrone. Scale bar: 50 μm.

**Figure:**
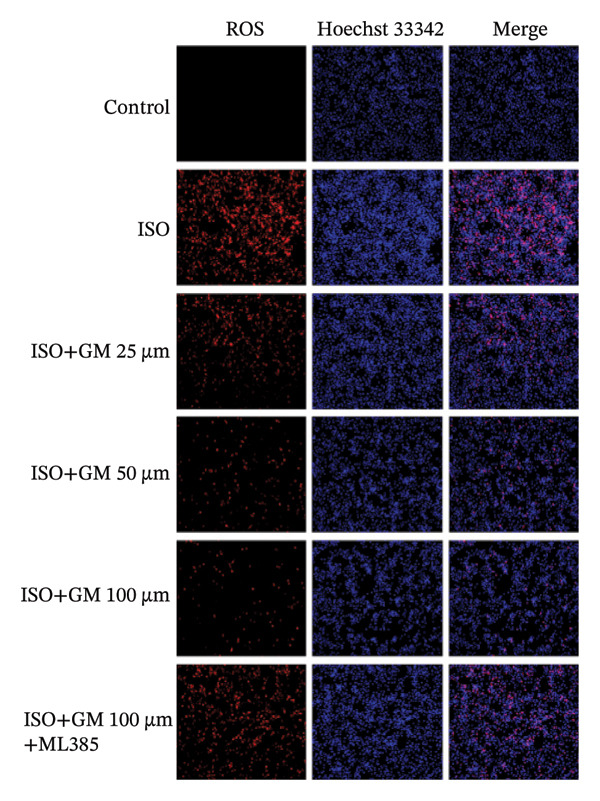
(a)

**Figure:**
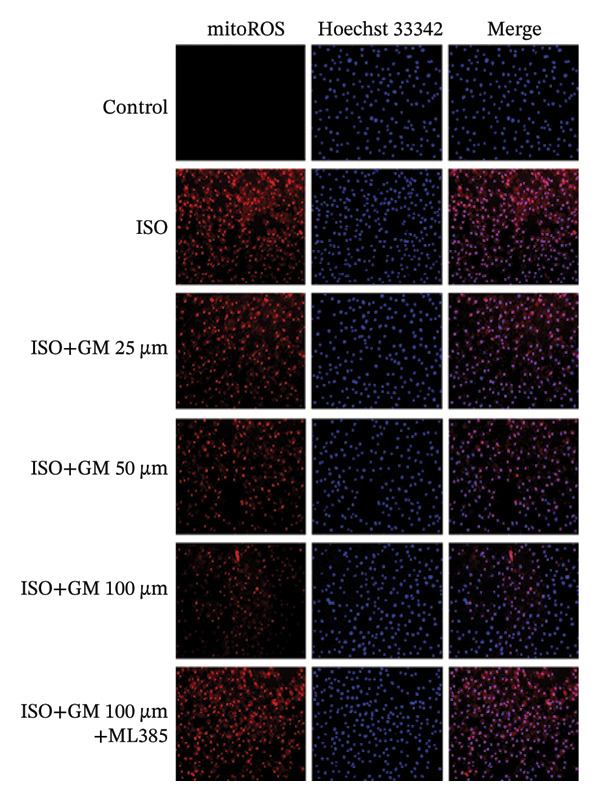
(b)

**Figure:**
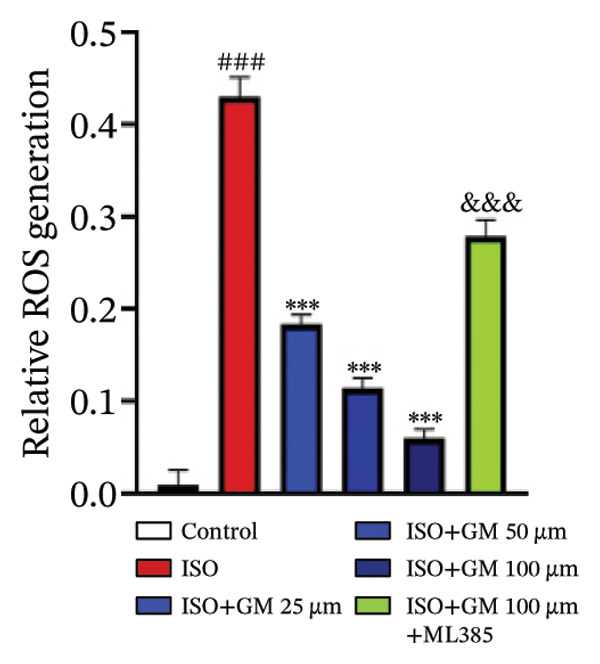
(c)

**Figure:**
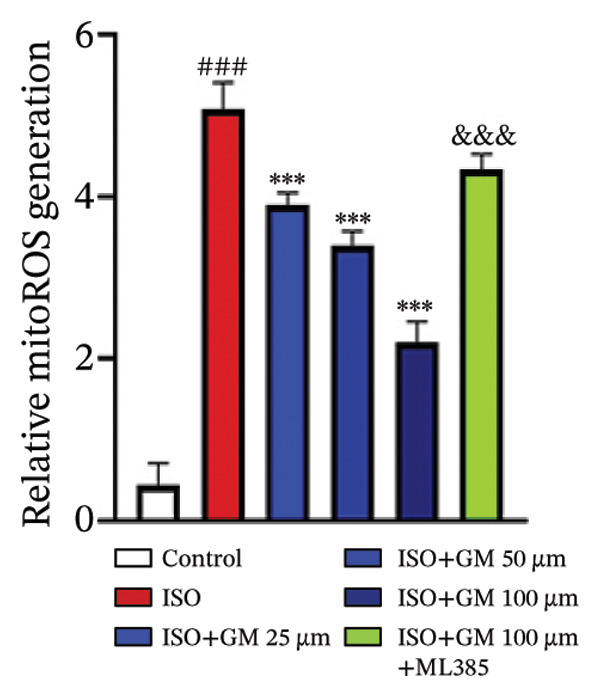
(d)

### 3.4. Germacrone Modulates Nrf2/GPX4 Signaling in Experimental Models

Nrf2 serves as a master regulator of oxidative stress responses and ferroptosis defense, coordinating the expression of key cytoprotective effectors including HO‐1, GPX4, and SLC7A11 through direct and indirect mechanisms. Integrative analysis across experimental models demonstrates germacrone’s capacity to counteract ISO‐mediated Nrf2 suppression while upregulating myocardial expression of the Nrf2/HO‐1/GPX4/SLC7A11 defense network (Figures 6(a), 6(b), 6(c), 6(d), 6(e), 6(f), 6(g), 6(h), 6(i), and 6(j)). These findings from our study substantiate that Nrf2 serves as a key target through which germacrone counteracts ISO‐induced myocardial ferroptosis. To further confirm germacrone’s impact on Nrf2, this study employed its inhibitor, ML385. The findings revealed that ML385 blocked germacrone’s ability to restore the decreased Nrf2, HO‐1, GPX4, and SLC7A11 protein expression levels caused by ISO. Collectively, these findings substantiate that germacrone induces pharmacological activation of the Nrf2/GPX4 signaling axis across both experimental models.

FIGURE 6Germacrone demonstrates Nrf2/GPX4 pathway modulation across experimental systems. (a–e) Germacrone‐mediated regulation of Nrf2/HO‐1/GPX4/SLC7A11 axis in murine myocardial tissue (*n* = 4). ^###^ *p* < 0.001 vs. control; ^∗^ *p* < 0.05, ^∗∗^ *p* < 0.01, ^∗∗∗^ *p* < 0.001 vs. ISO; ^&&^ *p* < 0.01, ^&&&^ *p* < 0.001 vs. high‐dose germacrone. (f–j) Proteomic modulation of Nrf2 pathway components (HO‐1, GPX4, and SLC7A11) by germacrone in H9c2 cardiomyocytes (*n* = 4). ^#^ *p* < 0.001 vs. control; ^∗∗^ *p* < 0.01, ^∗^ *p* < 0.001 vs. ISO; ^&&^ *p* < 0.01, ^&&&^ *p* < 0.001 vs. high‐dose germacrone.

**Figure:**
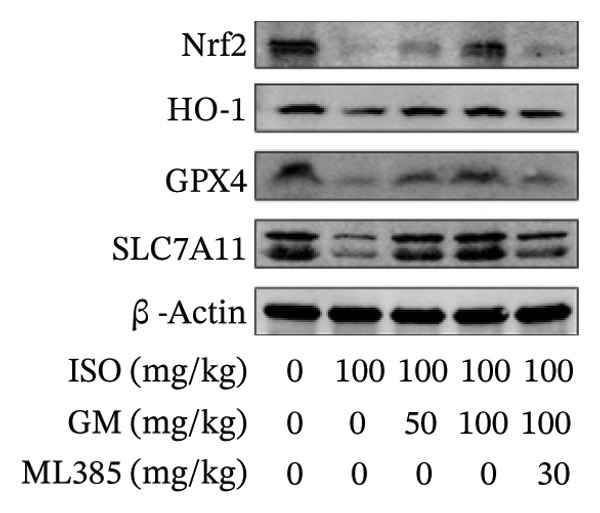
(a)

**Figure:**
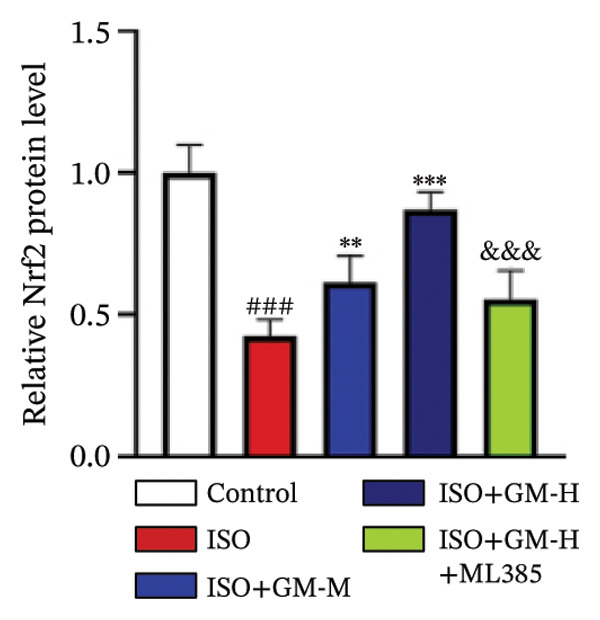
(b)

**Figure:**
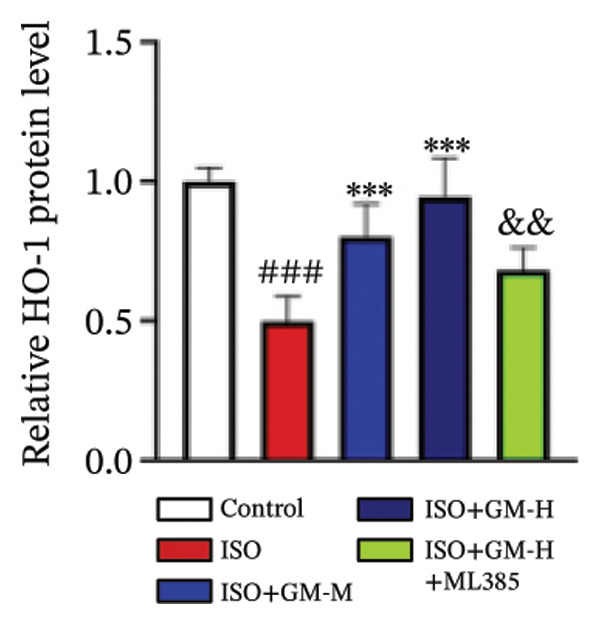
(c)

**Figure:**
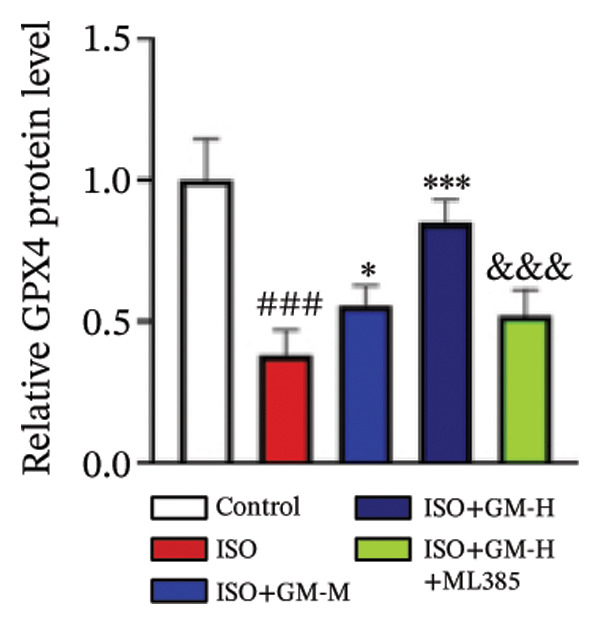
(d)

**Figure:**
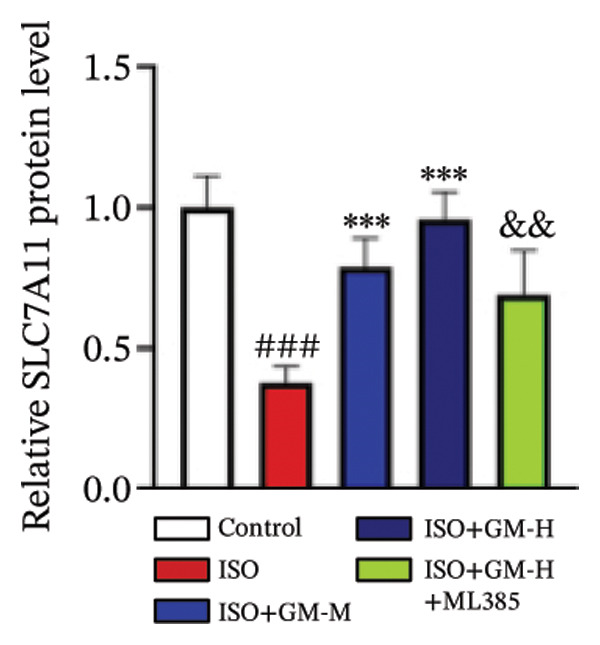
(e)

**Figure:**
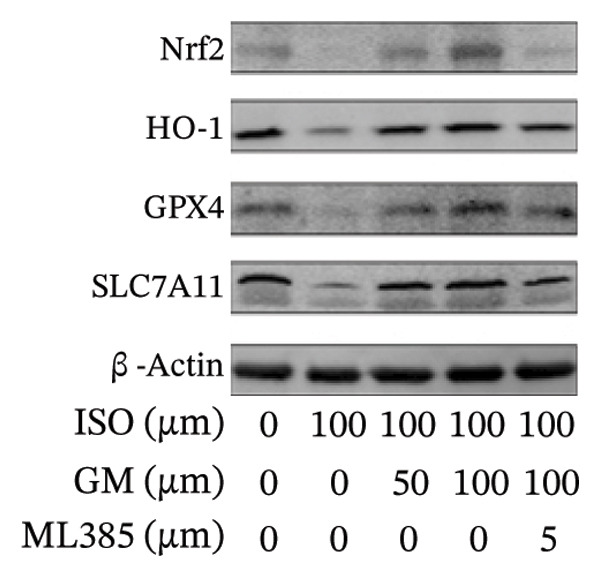
(f)

**Figure:**
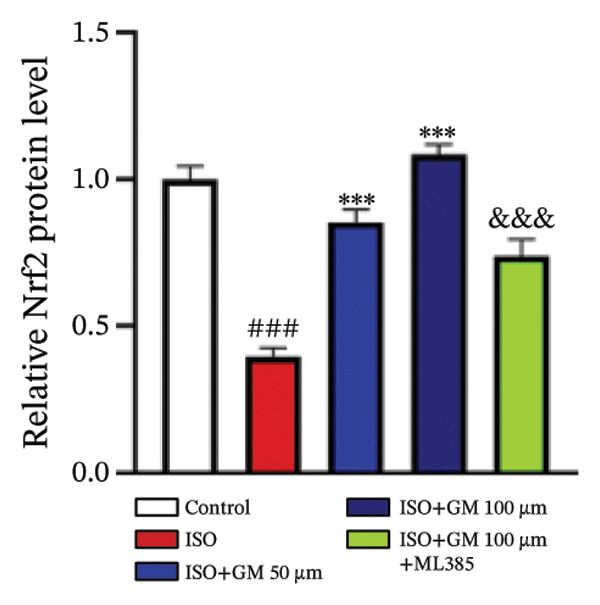
(g)

**Figure:**
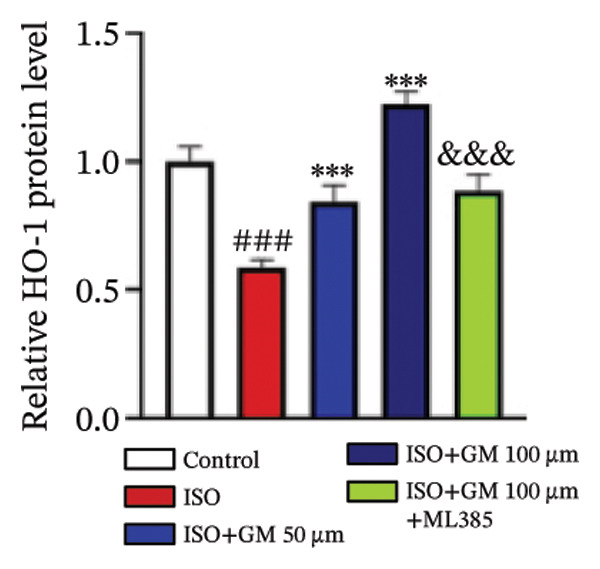
(h)

**Figure:**
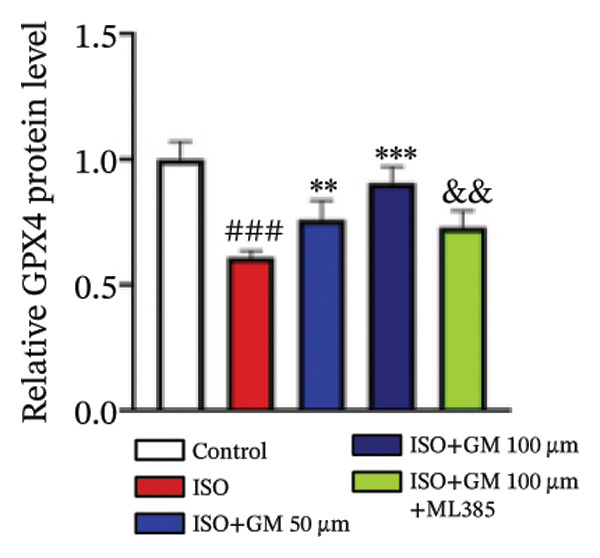
(i)

**Figure:**
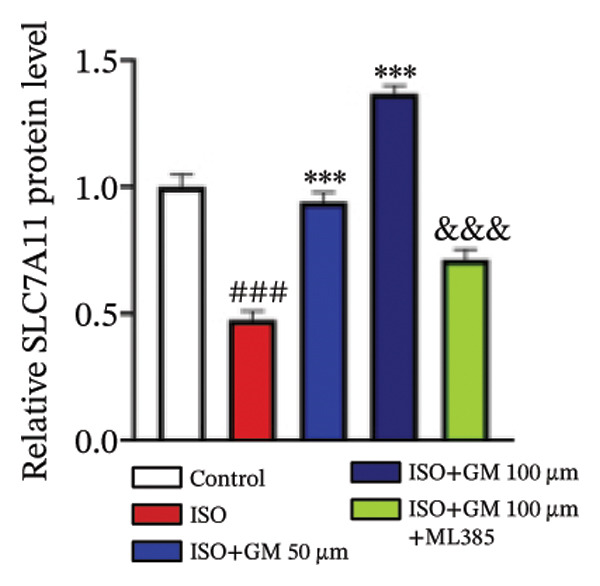
(j)

## 4. Discussion

MI remains the top cause of death among cardiovascular diseases globally, boasting an acute mortality rate as high as 30%. Acute coronary artery occlusion causes irreversible damage to myocardial cells and often results in life‐threatening complications such as ventricular fibrillation and cardiogenic shock [19]. Standard pharmacological treatments for MI currently include antiplatelet agents, lipid‐lowering drugs, beta‐blockers, angiotensin‐converting enzyme inhibitors, angiotensin receptor blockers, thrombolytics, analgesics, and sedatives [20]. Emerging therapeutic strategies [21] involve novel anti‐inflammatory agents like colchicine, IL‐6 receptor antagonists, and angiotensin IV, as well as advanced modalities such as stem cell therapy, gene therapy, and interventions targeting myocardial fibrosis. Despite these advances, current therapies have limitations, underscoring the ongoing clinical challenge of effectively managing MI. Therefore, exploring new therapeutic agents to complement existing treatment protocols is of significant clinical and scientific importance. This study substantiated, through both in vivo and in vitro experiments, that germacrone offers protective effects against ISO‐induced myocardial injury via suppressing myocardial ferroptosis. Additionally, germacrone‐mediated Nrf2/GPX4 pathway modulation may underlie this mechanism.

Germacrone, a natural sesquiterpene compound, possesses well‐documented anti‐inflammatory and antioxidant activities. Although numerous studies have reported its pharmacological activities, no evidence of its cytotoxicity in H9c2 cells has been documented [22]. Our study systematically evaluated the cytotoxicity of germacrone in H9c2 cells. The findings revealed that germacrone showed no cytotoxic effects at concentrations below 100 μM, while significant cytotoxicity was observed at 200 μM. Valsartan is widely recognized for its effectiveness in reducing the risk of myocardial reinfarction after PCI by enhancing cardiac function in patients with MI. Additionally, it decreases the occurrence of hospitalizations due to heart failure by preventing ventricular remodeling. Clinical data also indicate that valsartan is associated with a low incidence of adverse cardiac events and drug‐related adverse reactions, establishing it as an important part of the pharmacological treatment for MI [23]. In our in vitro experiments, valsartan was used as the positive control. Our results indicated that 100 μM of germacrone had a therapeutic effect similar to that of valsartan.

Previous studies have shown that germacrone can improve renal injury in diabetic mice by suppressing ferroptosis and podocyte apoptosis [24]. Notably, the potential cardioprotective role of germacrone in MI remains unexplored. To explore this, both animal and cellular experimental systems were used to establish an MI model. The results indicated that germacrone significantly improved cardiac function in mice, shown by dose‐dependent increases in EF and FS, together with decreases in LVEDV and LVESV. Additionally, germacrone treatment attenuated ISO‐induced myocardial histopathological alterations and substantially decreased circulating levels of cardiac damage markers (CK‐MB, LDH, and cTn‐I) in both animal and cellular experimental systems. These results establish germacrone as a promising therapeutic candidate for mitigating cardiac damage in MI.

The inflammatory cascade is a sequential process of activation and amplification mediated by multiple inflammatory factors, playing a key role in the initiation, progression, and clinical outcomes of MI [25]. MI often begins with a state of chronic inflammation, followed by a complex interaction between acute and chronic inflammatory responses [26]. Currently, various anti‐inflammatory agents are used in managing MI. Growing evidence supports the idea that targeting inflammation is a promising therapeutic strategy for improving outcomes in MI cases [27]. Apoptosis has been identified as a crucial mediator in the pathophysiological processes of MI and is increasingly seen as an important therapeutic target for improving patient prognosis [28]. Our results show that germacrone treatment markedly suppressed proinflammatory cytokine production (TNF‐α, IL‐1β, and IL‐6) across both animal and cellular experimental platforms. Additionally, germacrone markedly decreased the rate of apoptosis in H9c2 cells. These experimental outcomes align with existing literature while providing novel mechanistic evidence for germacrone’s dual anti‐inflammatory and antiapoptotic activities.

Ferroptosis represents a form of programmed cell death driven by iron‐dependent lipid peroxidation, contributing to impaired cardiac function and cardiomyocyte demise. Inhibiting ferroptosis has been shown to significantly reduce MI [29]. As an end‐product derived from lipid peroxidation cascades in biological systems, MDA represents a principal biomarker for assessing oxidative stress status. Elevated levels of MDA directly reflect the extent of cell membrane damage [30]. mtROS are the main components of cellular ROS and can work together with cytoplasmic ROS to promote the initiation, progression, and execution of ferroptosis [31]. The organisms’ antioxidant defense indicators include SOD, GPX, GSH, carotenoids, coenzyme Q, thioredoxin, and others [32]. Therefore, developing drugs targeting the ferroptosis pathway for MI treatment holds great potential. However, it remains unclear whether germacrone protects myocardial cells from MI by inhibiting this pathway. In the present study, germacrone administration significantly attenuated intracellular iron deposition and MDA content while concurrently enhancing GSH and SOD activities in both animal and cellular models of ISO‐induced myocardial injury. In H9c2 cells, germacrone was effective in inhibiting ROS and mtROS production. The findings support earlier research emphasizing the essential role of ferroptosis in the pathogenesis of MI and are particularly notable as the first demonstration that germacrone can reduce MI injury by blocking the ferroptosis pathway.

During ferroptosis in MI, ROS has been observed to promote the release of Nrf2 through the Nrf2/Keap1 system. After activation, Nrf2 translocates to the nucleus and specifically binds to antioxidant response elements, thereby inducing the expression of key genes such as HO‐1, GPX4, and SLC7A11 [33]. As shown in previous studies, *Salvia miltiorrhiza* inhibits ferroptosis in myocardial cells following MI by activating the Nrf2 signaling pathway [34]. The nuclear translocation of Nrf2 is a critical regulatory step that limits GPX4 transcription, thus promoting the occurrence, development, and progression of ferroptosis [35]. Therefore, ferroptosis, governed by the Nrf2 signaling pathway, has become a key new mechanism underlying the pathogenesis of MI. However, the cardioprotective effect of germacrone against MI via regulation of the Nrf2/GPX4 pathway remains to be clarified. This study shows that germacrone significantly promotes Nrf2 activation and concurrently increases the protein expression levels of HO‐1, GPX4, and SLC7A11. Furthermore, Nrf2 inhibitors markedly impede the germacrone‐induced elevation of Nrf2, HO‐1, GPX4, and SLC7A11 protein expression. These findings reinforce the view that the Nrf2/GPX4 pathway is a crucial target for regulating ferroptosis in myocardial cells and improving MI. Additionally, they clearly depict the Nrf2/GPX4 pathway as the key molecular mechanism through which germacrone provides its protective effects in MI.

While this study did not directly assess the distribution profile of germacrone in cardiac tissue, its systemic disposition and potential tissue accessibility are supported by existing pharmacokinetic and pharmacodynamic evidence. A validated HPLC method has confirmed that germacrone enters the systemic circulation in rats following oral or intravenous administration, indicating its systemic exposure [36]. More critically, in an ISO‐induced myocardial injury model, oral administration of germacrone significantly improved cardiac function, attenuated myocardial fibrosis, and downregulated levels of oxidative stress and inflammatory markers within cardiac tissue [16]. These pharmacodynamic outcomes strongly suggest that germacrone, or its active metabolites, can be effectively delivered to the myocardium to exert local biological effects. Collectively, this evidence supports the premise that germacrone possesses favorable cardiac tissue accessibility. Future studies should employ techniques such as radiolabeled tracing, mass spectrometry imaging, and tissue pharmacokinetic analysis to directly quantify the distribution and dynamic concentrations of germacrone in myocardial tissue, thereby systematically elucidating its cardiac‐targeted delivery and distribution characteristics. Regarding the vehicle used in this study, germacrone was dissolved in corn oil containing 0.4% DMSO for oral administration. The corn oil vehicle may enhance the oral bioavailability of this highly lipophilic compound by delaying gastric emptying, stimulating bile secretion, and promoting lymphatic absorption, thereby improving its intestinal solubility and absorption efficiency [37]. The observed significant improvement in cardiac function confirms that this vehicle effectively delivers the active compound to cardiac targets. We acknowledge that the lack of a comparison with different vehicle control groups is a limitation of the present study; future pharmacokinetic investigations are warranted to further delineate the specific contribution of the vehicle formulation.

MI is not only an acute injury event but also a major initiating factor in the progression toward chronic ventricular dysfunction and heart failure [38]. Oxidative stress, ferroptosis, and impaired redox signaling are increasingly recognized as key contributors to postinfarction remodeling [39]. Given the central role of Nrf2 in regulating myocardial redox homeostasis, mitochondrial integrity, and long‐term structural remodeling [40], the present finding that germacrone activates the Nrf2/GPX4 axis to suppress ferroptosis suggests that its protective effects may extend beyond acute cardiomyocyte survival, with potential implications for ventricular remodeling and heart failure progression. Early intervention targeting ferroptosis through this mechanism holds promise for attenuating ventricular dilation and delaying the onset of heart failure. Future studies employing chronic MI models are warranted to further validate this hypothesis.

## 5. Conclusion and Perspectives

This study confirms that germacrone alleviates myocardial injury following infarction by inhibiting ferroptosis via activation of the Nrf2/GPX4 pathway, elucidating its cardioprotective role and therapeutic potential, and providing a theoretical and experimental foundation for ferroptosis‐targeted cardiovascular therapy. Several limitations remain: The upstream molecular mechanisms by which germacrone activates Nrf2—such as potential interactions with Keap1 or modulation of Nrf2 nuclear translocation—are not fully understood; the research employed only an ISO‐induced acute injury model, which insufficiently replicates the chronic progression and long‐term ventricular remodeling of human MI; and the pharmacokinetics, bioavailability, and long‐term safety profile of germacrone have yet to be systematically evaluated. Future studies should validate its long‐term efficacy and impact on ventricular remodeling in more clinically relevant chronic MI models, further clarify its molecular targets and metabolic characteristics, and thereby provide a stronger scientific basis for advancing its clinical translation.

## Author Contributions

Jiaxiang Liao, Zitian Wang, and Zhou Huang were jointly responsible for the research design, data collection, and initial draft development.

Jincheng Li, Jie Yang, and Chunyun Fang contributed to data analysis and interpretation.

Dongling Huang, Fan Wang, and Xueling Lu participated in patient management and data acquisition.

Yiling Zhai substantially enhanced the manuscript’s intellectual rigor through critical revisions and holds correspondence duties.

Wei Wang supervised the entire study, provided clinical guidance, and is the corresponding author.

## Funding

This work was funded by the National Natural Science Foundation of China (No. 81860346) and the Guangxi University Key Laboratory of Emergency Medicine.

## Disclosure

All authors gave final approval to the manuscript version for publication and accept full accountability for all aspects of the research.

## Ethics Statement

Prior to initiation of this research, ethical approval (No. 2025‐D0457) was secured from the Institutional Ethics Committee of the First Affiliated Hospital of Guangxi Medical University for all study protocols. The approval document of the ethics committee is in the supporting information.

## Consent

All enrolled individuals provided written informed consent before any study procedures were conducted.

## Conflicts of Interest

The authors declare no conflicts of interest.

## Supporting Information

Additional supporting information can be found online in the Supporting Information section.

## Supporting information


**Supporting Information** Supporting Figure S2. Schematic diagram of the experimental timeline. Mice underwent echocardiography 24 h after the final ISO injection, followed immediately by euthanasia and cardiac tissue collection.

## Data Availability

The datasets generated in this investigation are archived by the authors and may be shared under stipulated conditions.
