# Supplementary figures and images for: Nrf2/GPX4‐Dependent Ferroptosis Inhibition: The Central Mechanism Underpinning Germacrone‐Induced Cardioprotection in Myocardial Infarction
Source: Adv Pharmacol Pharm Sci. 2026 May 26;2026:7893262. doi: 10.1155/adpp/7893262 (PMC13202446; doi:10.1155/adpp/7893262)

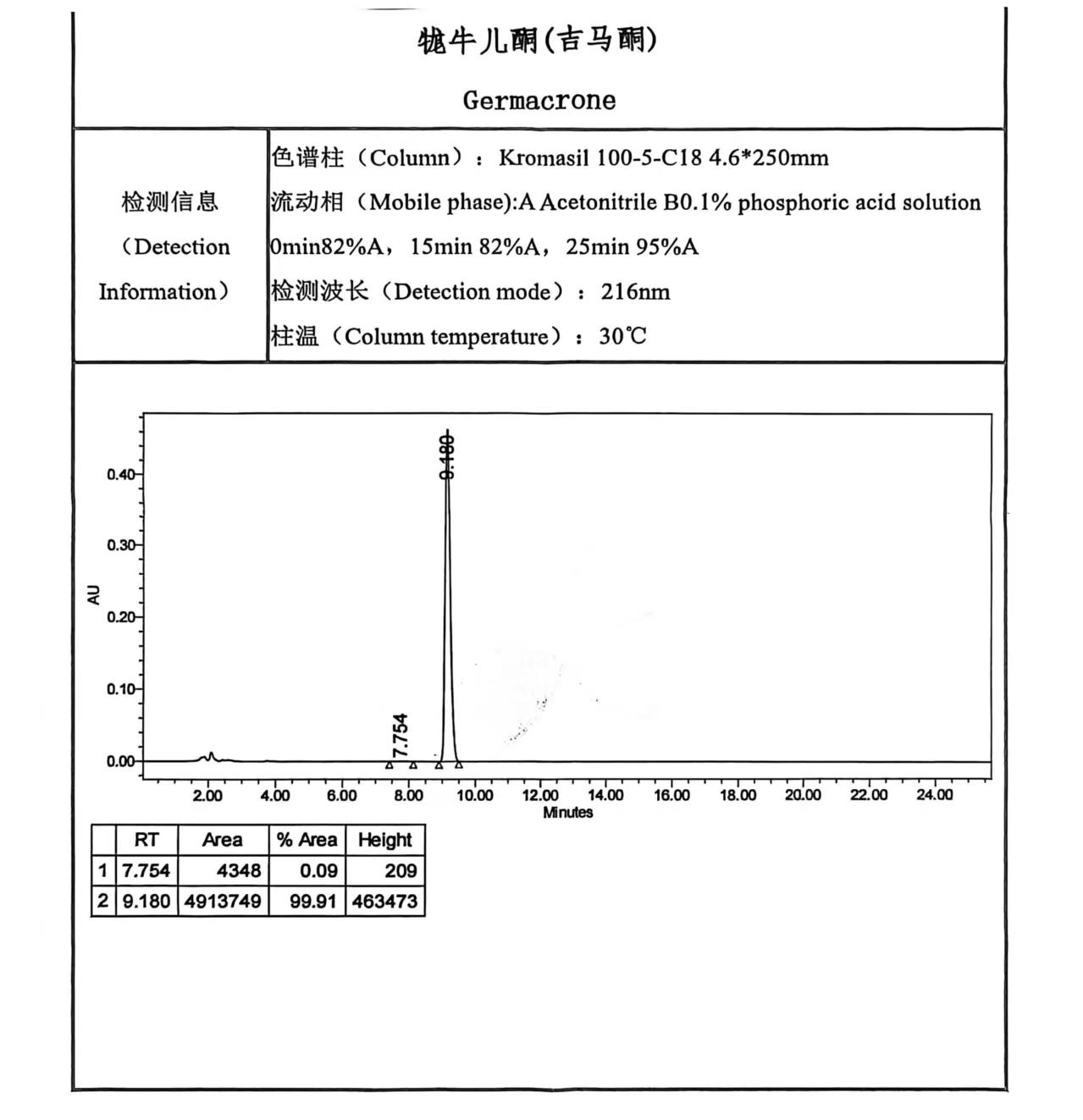

Supplement: Supplementary file 1 — Supporting Information Supporting Figure S2. Schematic diagram of the experimental timeline. Mice underwent echocardiography 24 h after the final ISO injection, followed immediately by euthanasia and cardiac tissue collection. [file ADPP-2026-7893262-s001.zip › Supplementary Figure S1.jpg]

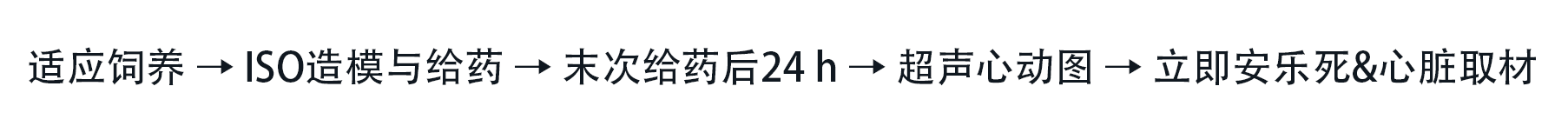

Supplement: Supplementary file 1 — Supporting Information Supporting Figure S2. Schematic diagram of the experimental timeline. Mice underwent echocardiography 24 h after the final ISO injection, followed immediately by euthanasia and cardiac tissue collection. [file ADPP-2026-7893262-s001.zip › Supplementary Figure S2.tif]
